# AZD9773, a novel anti-TNFα immune Fab in development for severe sepsis and septic shock: demonstration of safety and efficacy in a murine CLP sepsis model

**DOI:** 10.1186/cc10410

**Published:** 2011-10-27

**Authors:** P Newham, P Ceuppens, S Das, JWT Yates, R Knight, JS McKay

**Affiliations:** 1 Global Safety Assessment, AstraZeneca, Macclesfield, Cheshire, UK; 2 Discovery Enabling Capabilities and Sciences, AstraZeneca, Macclesfield, Cheshire, UK; 3 Clinical Pharmacology & DMPK, AstraZeneca, Macclesfield, Cheshire, UK; 4 Oncology iMed DMPK, AstraZeneca, Macclesfield, Cheshire, UK

## Introduction

TNFα is thought to play a central role in the pathogenesis of sepsis and septic shock. AZD9773 is an ovine polyclonal anti-human TNFα immune Fab comprising TNFα-directed and nonspecific Fab populations. AZD9773 potency and pharmacokinetic attributes such as a shorter half-life distinguish it from anti-TNF monoclonal antibodies, which have been assessed previously in clinical sepsis models. Here we explore the preclinical safety/efficacy of AZD9773 in a mouse cecal ligation puncture (CLP) model. There are currently no reports of anti-TNF agent efficacy in mouse CLP; rather, TNFα neutralization is associated with minimal/no effect or increased mouse mortality. As AZD9773 is differentiated from other anti-TNFα agents based on neutralizing potency and pharmacokinetic attributes, we studied both the safety and efficacy of this product in mouse CLP models.

## Methods

We studied AZD9773 (plus imipenem) effects in two mouse CLP models: a mild-grade model to explore the potential for AZD9773 to compromise mouse survival, and a severe-grade model to test AZD9773 efficacy. CLP (mild-grade sepsis) comprised 100% cecal ligation and single 20-gauge needle puncture, while CLP (severe-grade sepsis) comprised 100% cecal ligation and single 18-gauge needle puncture. Saline resuscitation and imipenem administration were included in both models. Since AZD9773 does not bind or neutralize murine TNFα, the CLP models were established in Tg1278/-/- (human TNFα transgene/mouse TNFα null) mice. An equivalent protein dose of DigiFab (plus imipenem) served as an irrelevant Fab control. Survival was monitored for 5 days.

## Results

The control severe-grade model resulted in approximately 20% survival at 5 days. Therapeutic i.p. dosing of AZD9773 bid from 24 to 60 hours (first dose 4,000 units/kg, second to fourth doses 2,000 units/kg) resulted in statistically significant increases in survival (>70%) compared with i.p. DigiFab control (*n *= 15 per group). The mild-grade model resulted in 63% survival with imipenem alone, 65% survival with AZD9773 and 69% survival with DigiFab at 5 days. Thus, therapeutic dosing of AZD9773 bid from 24 to 60 hours (schedule/dose/route as previously) did not result in significantly different survival outcomes versus either DigiFab or imipenem alone (*n *= 60 per group).

## Conclusion

These data demonstrate for the first time that TNFα neutralization in a murine CLP model improves survival in a severe sepsis setting. Moreover, contrasting with previous reports, TNF suppression in mild-grade CLP models is not associated with increased mortality. These findings support the hypothesis that AZD9773 has potential to be differentiated from other anti-TNF agents as a therapeutic intervention in sepsis.

## Conflicts of interests

All authors are employees of AstraZeneca.

